# Which Has a Greater Impact on Plant Functional Traits: Plant Source or Environment?

**DOI:** 10.3390/plants13060903

**Published:** 2024-03-21

**Authors:** Ling Xian, Jiao Yang, Samuel Wamburu Muthui, Wyckliffe Ayoma Ochieng, Elive Limunga Linda, Junshuang Yu

**Affiliations:** 1 Core Botanical Gardens/Wuhan Botanical Garden, Chinese Academy of Sciences, Wuhan 430074, China; xianling@wbgcas.cn (L.X.); smuthui41@gmail.com (S.W.M.); ochiengwycliffe5@gmail.com (W.A.O.); 2 School of Life Sciences, Hubei University, Wuhan 430062, China; 18287240625@163.com; 3 Sino-Africa Joint Research Centre, Chinese Academy of Sciences, Wuhan 430074, China; 4 University of the Chinese Academy of Sciences, Beijing 101408, China; 5 School of Resources and Environmental Science, Hubei University, Wuhan 430062, China; limungaelive@gmail.com; 6 Changjiang Water Resources and Hydropower Development Group Co., Ltd., Wuhan 430010, China

**Keywords:** adaptive strategy, plant functional trait, plant trait network, freshwater ecosystem restoration, submerged macrophytes

## Abstract

The deterioration of water quality caused by human activities has triggered significant impacts on aquatic ecosystems. Submerged macrophytes play an important role in freshwater ecosystem restoration. Understanding the relative contributions of the sources and environment to the adaptive strategies of submerged macrophytes is crucial for freshwater restoration and protection. In this study, the perennial submerged macrophyte *Myriophyllum spicatum* was chosen as the experimental material due to its high adaptability to a variable environment. Through conducting reciprocal transplant experiments in two different artificial environments (oligotrophic and eutrophic), combined with trait network and redundancy analysis, the characteristics of the plant functional traits were examined. Furthermore, the adaptive strategies of *M. spicatum* to the environment were analyzed. The results revealed that the plant source mainly influenced the operational pattern among the traits, and the phenotypic traits were significantly affected by environmental factors. The plants cultured in high-nutrient water exhibited a higher plant height, longer leaves, and more branches and leaves. However, their physiological functions were not significantly affected by the environment. Therefore, the adaptation strategy of *M. spicatum* to the environment mainly relies on its phenotypic plasticity to ensure the moderate acquisition of resources in the environment, thereby ensuring the stable and efficient operation of plant physiological traits. The results not only offered compelling evidence on the adaptation strategies of *M. spicatum* in variable environments but also provided theoretical support for the conservation of biodiversity and sustainable development.

## 1. Introduction

With the rapid development of agriculture, industrialization, and urban construction, environmental deterioration caused by human activities, such as agricultural non-point source pollution, industrial wastewater, and urban sewage discharge, has become a significant factor affecting ecosystems and human life [1,2]. The excessive inputs of nutrients in water bodies have led to the serious disruption of ecosystems’ structure and function, resulting in severe damage to freshwater ecosystems [3,4].

Submerged macrophytes are one of the primary components in freshwater ecosystems. They not only suppress sediment suspension and provide spawning and refuge habitats for aquatic animals but also effectively alleviate the nutrient load of water bodies through absorption and utilization, playing a crucial role in maintaining the structure and function of the ecosystem [5,6,7]. However, the decline of submerged macrophytes has occurred in recent years due to the deterioration of water quality [8,9,10]. Nevertheless, a few species demonstrate a strong adaptability to withstand harsh environments and maintain normal growth and reproduction. These species are suitable pioneer candidates in freshwater ecosystem restoration. Therefore, it is essential to explore the strategies of submerged macrophytes in adapting to a variable environment, which also play a crucial role in the protection and restoration of water resources.

The plant adaptability is related to the capability of plants to survive and thrive under various environment conditions, often manifested through alterations in plant functional traits [11]. Plant functional traits, including morphology, anatomical structure, and physiological characteristics, are exhibited throughout the entire lifespan of plants [12,13]. They determine the manner in which plants interact with the external environment directly. Plant functional traits can be adjusted to enhance their survival and reproductive capability in specific environments through perception and response to environmental factors. For example, in a low-light environment, plants may develop a larger leaf area to promote photosynthetic efficiency [14]. Changes in plant functional traits are also vital for preserving biodiversity and ecosystem stability [11,15]. Plants with high adaptability can quickly adjust their functional traits when the environment changes, maintaining the basic function of the ecosystem and providing habitats for the survival of other organisms, thereby supporting the coexistence of more species.

Plant functional traits are often influenced by multiple factors, among which the environment and plant source have attracted the most attention from researchers [7,16,17,18,19,20]. The environment has a direct and significant impact on plant functional traits, leading to changes in the plant morphology, physiology, and ecology [21,22,23]. Yuan et al. [7] found that when resources are limited, plants will replenish starch by breaking down their stored reserves. Additionally, they will adjust the distribution of biomass between tissues, increasing the plant height in order to acquire more photosynthetic carbon. Wei et al. [23] conducted a warming experiment on four dominant species in the permafrost ecosystem of the Qinghai–Tibetan Plateau. Their findings revealed that warming led to a change in community functional traits, favoring more acquisitive characteristics. These included earlier green-up, taller plants, larger leaves, increased photosynthetic resource use efficiency, thinner roots, and higher values for specific root length and root nutrient concentrations. However, the impact on the functional diversity was found to be insignificant. The plant source is also an important factor determining the plant’s functional traits. In this current investigation, “plant source” refers to the geographical location from where the plant materials were obtained. Plants from different sources may accumulate various genetic and adaptive characteristics during their evolution, directly affecting plant functional traits and adaptability [20,24]. However, most previous research has only focused on one single aspect in various factors or the interactions of multiple environmental factors [7,17,18,19]. The question of whether the environment or plant source has a more significant influence on determining plant functional traits remains unanswered.

In this study, populations of *M. spicatum* were chosen as materials because of its high adaptability to harsh environments [25,26,27,28]. Despite being an invasive species in some parts of the world, it is an ideal ecological restoration species in China and countries where it is not considered invasive. Reciprocal transplant experiments in two different artificial environments (oligotrophic and eutrophic) were conducted: Sampling shoots were collected from two lakes with different trophic levels and transplanted into artificial freshwater ecosystems and cultured separately using water from the two lakes. Long-term observation was conducted, and plant functional traits were measured. This study aimed to compare the importance of the plant sources and environment in determining the plant functional traits. It not only provides appropriate evidence for comprehending the strategies of plant adaptation to environment, contributing to the cultivation of new candidates in freshwater restoration, but also provides theoretical support for biodiversity protection in sustainable development.

## 2. Results

### 2.1. Overall Topological Structure of Network

**Figure 1:**
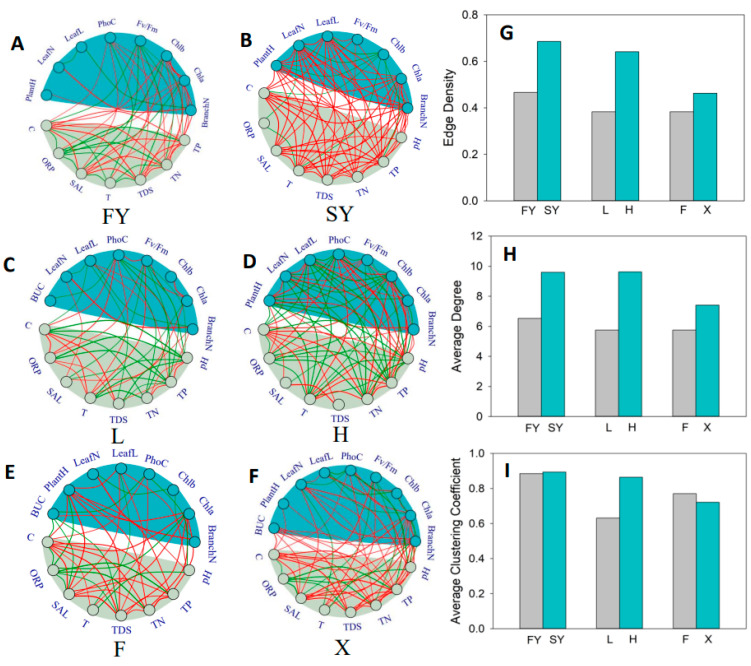
Effect of culture time point, culture medium, and plant source on trait network. The subplots (**A**–**F**) show the trait networks for different treatments and correlations among traits (indicated by color and edge thickness). Red edges indicate positive correlations, while green edges indicate negative correlations between the two connected traits. The subplots (**G**–**I**) show the network parameters, i.e., edge density, average degree, and average clustering coefficient of different treatments. The abbreviations in figures are explained as follows: PlantH—plant height, BranchN—branch number, LeafL—leaf length, LeafN—leaf number, Fv/Fm—maximum quantum efficiency of PSII photochemistry, Chla—chlorophyll-a content, Chlb—chlorophyll-b content, PhoC—photosynthetic capacity, BUC—bicarbonate utilization capacity, C—conductivity, ORP—oxidation reduction potential, SAL—salinity, T—temperature, TDS—total dissolved solids, TP—total phosphorus, and TN—total nitrogen.

The Network analysis showed that the structure of the network topology changed significantly in different treatments. We compared the topological structures of the two culture time points (FY and SY) (Figure 1A,B). The edge density of SY was about 46.81% higher than FY and the average degree of SY was about 47.01% higher than FY (Figure 1G,H). The average clustering coefficient of the two culture time points did not show much difference (Figure 1I). Similar trends of the edge density and average degree were observed in the networks of L nutrition and H nutrition (Figure 1C,D). The edge density and average degree were about 68.42% and 67.48% higher in H nutrition than L nutrition, respectively (Figure 1G,H); however, the value of the average clustering coefficient of H nutrition was still higher (about 36.51%) than L nutrition (Figure 1I). We further compared the topological structures of the network constructed with the samples from F source with the network of X (Figure 1E,F). Both values of the edge density and average degree in the network of X were much higher than those of F; they were about 21.05% and 28.87% higher, respectively. (Figure 1G,H). However, the average clustering coefficient of X was 6.49% lower than that for F (Figure 1I).

### 2.2. Correlation Analyses of Plant Traits with Environmental Factors

**Figure 2:**
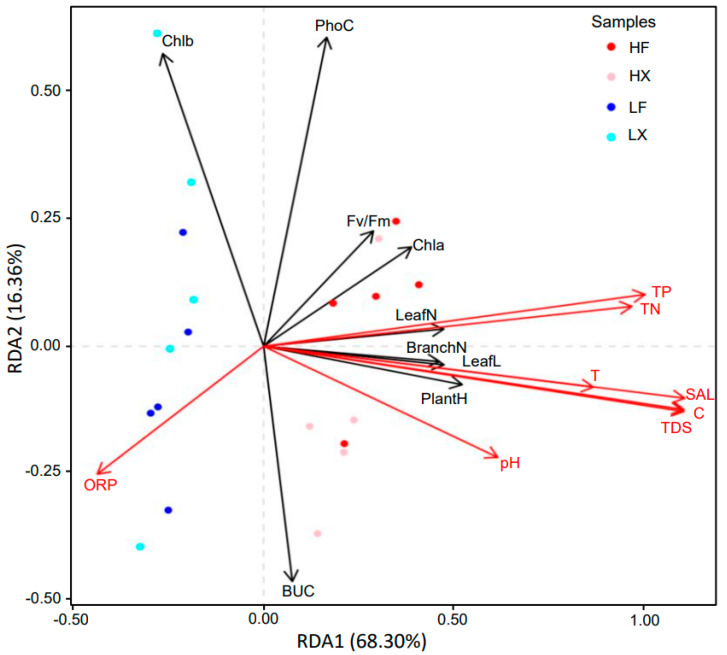
Redundancy analysis (RDA) of samples. The points with different colors represent different treatments. The abbreviations are the same as in Figure 1.

The redundancy analysis (RDA) was applied to identify the explanation of environmental variables to the plant traits, including the plant height, branch number, leaf length, leaf number, Fv/Fm, chl *a*, chl *b*, photosynthetic capacity, and bicarbonate utilization capacity. Considering the adaptation of plants to their habitat, we focused on the data from the second year (SY) of cultivation. The RDA showed that the environmental explanatory variables can explain approximately 74.66% of the variability of the samples, in which RDA1 explained 68.30%, while RDA2 explained 16.36% of the variability (Figure 2). The Mantel analyses showed that C (r = 0.7344, *p* = 0.001), SAL (r = 0.7291, *p* = 0.001), TDS (r = 0.7265, *p* = 0.001), TP (r = 0.604, *p* = 0.001), TN (r = 0.6103, *p* = 0.001), and T (r = 0.4790, *p* = 0.001) had significant correlations with plant functional traits; however, the pH and ORP had no significant relationships (*p* > 0.05). The plant traits related to morphology, such as the plant height, leaf length, leaf number, and branch number had a significant positive correlation with environmental factors including TP, TN, SAL, T, TDS, and C. However, the plant traits related to physiology, such as Fv/Fm, chl *a*, chl *b*, photosynthetic capacity, and bicarbonate utilization capacity had a lower correlation with the environmental factors.

### 2.3. Changes in Plant Phenotypic Plasticity

**Figure 3:**
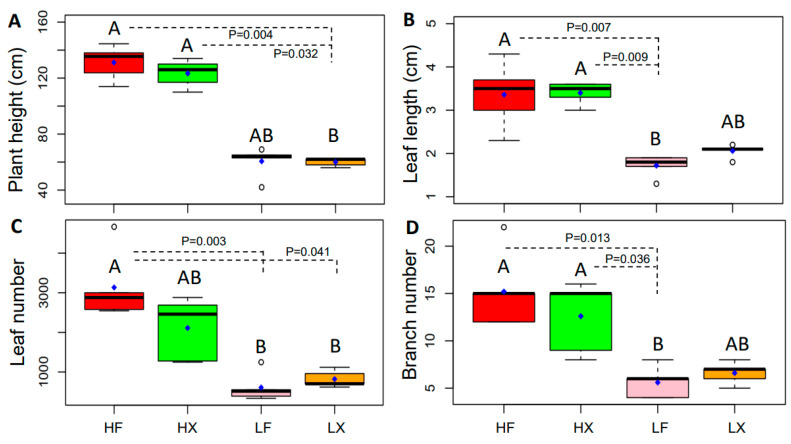
Characteristics of plant height (**A**), leaf length (**B**), leaf number (**C**), and branch number (**D**) of *Myriophyllum spicatum* in the second year of cultivation of different treatments. The box in the plot represents the interquartile range (IQR), which contains the middle 50% of the data. The line inside the box denotes the median, while the blue point signifies the mean value of the dataset. The “whiskers” extending from the box show the range of the data, excluding outliers. Outliers are represented as individual points beyond the whiskers. Different letters indicate significant differences (*p* < 0.05) after the Kruskal–Wallis test.

To compare the characteristics of the plant functional traits in different treatments, a Kruskal–Wallis test and post hoc multiple comparisons were performed. The samples cultivated in water from Xingyun Lake (X) had a relatively higher plant height (Figure 3A). The plant height of the samples from HF and HX was approximately 131.16 and 123.40 cm, respectively, while that of the samples from LF and LX was about 60.60 and 60.00 cm, respectively. The leaf length, leaf number, and branch number also exhibited a similar tendency with the plant height (Figure 3B–D). The leaf length of the samples from HF and HX was about 3.36 and 3.40 cm, and only 1.72 and 2.06 cm for the samples from LF and LX, respectively (Figure 3B). The leaf number of the samples from HF and HX was about 3133.60 and 2110.80, and only 608.80 and 820.80 for the samples from LF and LX, respectively (Figure 3C). Similarly, the branch number of the samples from HF and HX was about 15.20 and 12.60, and only 5.60 and 6.60 for the samples from LF and LX, respectively (Figure 3D). There were no significant differences in the plant physiological trait indicators: Fv/Fm (*p* = 0.077), PhoC (*p* = 0.182), BUC (*p* = 0.054).

## 3. Material and Methods

### 3.1. Experimental Site and Plant Material

**Figure 4:**
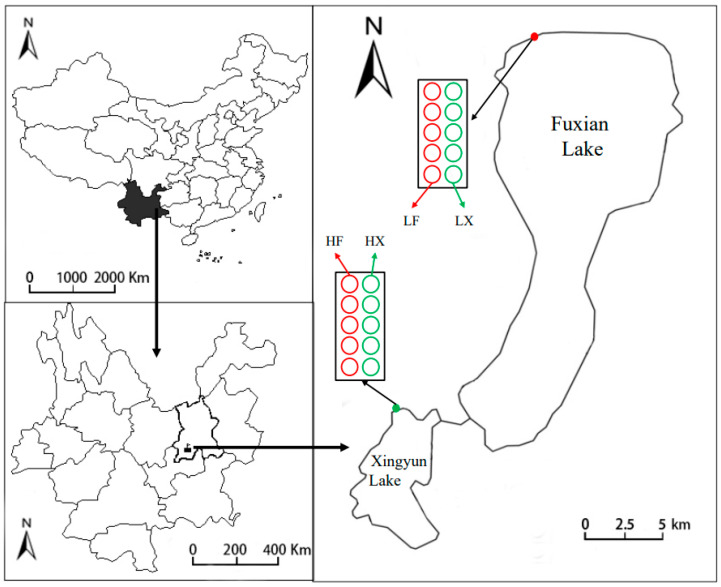
Location of Fuxian Lake and Xingyun Lake in Yunnan Plateau, Southwest China, and reciprocal transplant design.

Materials in this study were collected from Fuxian and Xingyun lakes. Both lakes are located in the center of Yunnan Plateau, Southwest China. Fuxian Lake (24.37°–24.63° N, 102.81°–102.95° E) is oligotrophic with clear conditions, while Xingyun Lake (24.33° N, 102.79° E) is eutrophic with heavy nutrient loading throughout the year [27,29]. The two lakes maintained healthy states in the early 1980s, whereas Xingyun Lake gradually transitioned to a eutrophic state and cyanobacterial blooms appeared almost every year because of the interference of human activities in recent decades. *Myriophyllum spicatum* dominates both Fuxian and Xingyun lakes. The close geographical location, together with the contrasting trophic conditions in the two lakes, make them favorable environments for exploring the adaptive strategies of species. Through comparative analysis of the plant responses to different water environments within the lakes, we can enhance our understanding of the precise impacts of water conditions on plant phenotypic traits. Transplant experiments were conducted at two study sites adjacent to the lakes to investigate the combined effect of nutrition/trophic status and plant source (Figure 4).

### 3.2. Plant Cultivation

In May 2014, healthy shoots of *M. spicatum* were collected from the same colony in Fuxian (labeled F, for individuals from Fuxian Lake) and Xingyun (labeled X, for individuals from Xingyun Lake) lakes, respectively. Ten-centimeter (10 cm) apices without leaves of the plants were cleaned and transplanted into plastic tanks (height 70 cm, diameter 53 cm, volume 160 L) filled with sediment and water from Fuxian (labeled L) and Xingyun (labeled H) lakes, respectively. Thus, 4 treatments (LF, LX, HF, and HX) were generated, which were placed outdoors near the lakes in well-lit areas. For each treatment, 5 repetitions were performed. Thus, 20 tanks were used and each tank had one individual plant. The water in plastic buckets was renewed every 3 days to maintain the homogeneity of the natural habitats. During the one-year cultivation period, the plants’ traits and water environmental indicators were regularly monitored. Water environmental indicators are shown in Table 1. Identical plants and detailed information of reciprocal transplants have been previously described in Xian et al. (2020) [29].

### 3.3. Estimation of Water Quality and Plant Traits

Parameters related to water quality, including conductivity (C), oxidation reduction potential (ORP), pH, salinity (SAL), temperature (T), total dissolved solids (TDS), total phosphorus (TP), and total nitrogen (TN), were measured in four treatments. TP and TN were measured using UV spectrophotometry [28,30]; the other parameters were measured using a multi-parameter water quality analyzer (YSI, Pro Plus).

Plant morphological traits, including plant height, branch number, leaf length, and leaf number, were recorded, and physiological parameters, including chlorophyll fluorescence (Fv/Fm), chlorophyll content (chl *a* and chl *b*), photosynthetic capacity, and bicarbonate utilization capacity, were assessed in August 2014 (the first year, FY) and 2015 (the second year, SY), respectively. Fv/Fm was determined with a PAM 2100 fluorometer (PAM-2100, Walz, Effeltrich, Germany), chlorophyll content was measured by extracting 0.1 g fresh leaves in ethanol for approximately 24 h in the dark; the absorbance was measured by spectrophotometer [27,28]. Photosynthetic capacity of plants was characterized through O_2_ evolution at 25 °C at a photon irradiance of 120 μmol photon m^−2^ s^−1^ according to the methods described in Zhang et al. (2013) [26]. The ability of leaves to use bicarbonate was measured according to Maberly and Spence (1983) [31].

### 3.4. Plant Trait Network Analysis

To decipher the relationship of plant traits and environment among treatments, plant trait network analysis combined with environmental factors was performed. In this network, plant traits and environmental factors are nodes, and the relationships between each indicator are edges. A matrix of relationships (r) between each node was calculated after normalized transformation for 17 indicators, including 8 plant traits and 9 environmental factors. Pearson correlations were used to quantify the relationships. A threshold of |r|> 0.2 indicated significant correlations among nodes at *p* < 0.05, which was used to avoid spurious correlations among traits [7,32,33]. The absolute value of Pearson correlation coefficients (|r|) was used to describe the strength of node–node relationships [34]. The package “igraph” of R version 4.3.0 was used to transform the matrix of relationships between each node into a network graph. To investigate the correlations among different traits in each treatment, 6 networks were constructed and named “FY”, “SY”, “L nutrition”, “H nutrition”, “F”, and “X”. Then, parameters of networks were calculated using the package “igraph”.

To explore characteristics of correlations among nodes in each network, three network topological properties were calculated: (1) “Edge density (ED)”, defined as the proportion of actual connections among nodes of all possible connections [35], it quantifies the tightness of the overall topology of the network, and can be used to describe the trade-off between the cost versus the efficiency of connections [19]. Networks with a higher value of ED mean that plants are able to acquire and mobilize resources efficiently; however, it may be costly in the establishment of the connections and the maintaining of relationships between traits [34]. (2) “Average clustering coefficient (AC)” describes the average of the clustering coefficients of all nodes in the network; it quantifies the complexity of the overall topology of network. A higher AC in a network means the network is more extensively divided into several different components [35]. (3) “Degree” is one of the parameters used to describe the properties of nodes within network, which can be used to qualify the connectedness of each node [35]. Degree is the number of edges that connect a focal node to other nodes, a higher degree means more importance of the specific traits in the community, which can be considered overall hub traits [34,36].

### 3.5. Statistical Analysis

To decipher the relationship between environment factors and plant traits, redundancy analysis (RDA) was conducted using the “vegan” package in R software version 4.3.0, and Mantel analysis was used to test the correlations of each environmental factors to plant functional traits through the function of “mantel”. The Kruskal–Wallis test and post hoc multiple comparisons test were applied to compare the differences in plant functional traits among treatments using SPSS software version 22.0. Visualization of figures was performed using the “ggplot2” package in R 4.3.0.

## 4. Discussion

### 4.1. The Topological Structure of the Trait Network

The network theory offers an effective approach to investigate relationships between multiple plant traits. It has been extensively applied in various analyses [35], such as metabolic pathways [37,38], gene interactions [39,40], microbial communities [41], and transport systems [42,43]. Trait networks possess the potential to comprehensively capture and visualize the associations among various traits. To provide more accurate descriptions on the topology structure of networks and the interdependency between traits, recent studies have quantified several parameters such as edge density, average clustering coefficient, and degree. Furthermore, ecological significance has been assigned to these parameters [34,36,44].

It is evident that as the culture time increases, the value of the edge density and average degree of the trait network increased significantly. A similar tendency could be observed in the treatments with different nutrients and sources. Both the value of the edge density and average degree were higher in H nutrition (compared with L nutrition) and X (compared with the source from F). The higher values of these parameters suggest that the topological structure of the trait networks becomes tighter and the connectivity among traits are stronger [7,34,44], which means that the relationships between the plant functional traits and environmental factors become closer. The correlations between traits are regarded as the trade-offs of resource allocation or synergistic optimization among traits to meet the basic demands of survival [7]. A higher connectivity means that numerous traits are significant in driving functioning, and the efficiency of resource transfer between traits is higher [7,36]. With an increase in the culture time, *Myriophyllum spicatum* showed a strong adaptability to its surroundings. Correspondingly, plants cultivated in high-nutrient environments (H nutrition) exhibited stronger abilities in resource allocation and synergistic optimization, like plants from X source (from a lake with a higher trophic level). This may be attributed to the partially surplus resources, such as nitrogen and phosphorus, despite the limitation of other factors like light in high-nutrient environments. In order to survive in complex environments, plants have gradually developed stable and efficient cooperative patterns for mobilizing nutrients and energy among different functional traits. These patterns enable plants to quickly adjust their functional traits and adapt to new environments more rapidly.

A relatively higher value of the average clustering coefficient (AC) was observed in the networks of H nutrition and F compared with L nutrition and X, respectively. The value of the AC represents the complexity of the network [34,35]. A higher AC value means that the networks are extensively divided into several different components [35]; it quantifies the extent of trait clustering or trait modularity [7,34,35]. A high average clustering coefficient suggests that functional traits within the plant community are strongly interconnected, forming clusters or modules, while the connections between modules are weak [7,32]. This indicates functional trait convergence or a tendency for plants with similar traits to coexist and interact more frequently [7,34,35]. In this study, the complexity of the trait network did not change a lot in different culture time points. The higher complexity observed in H nutrition and F indicated a higher modularity of the two networks, which means that the modules composed of specific traits perform specific functions. Plants with more modules will experience more flexibility and freedom to respond to unfavorable environments [7,32]. Therefore, *M. spicatum* in the H nutrition environment had more flexibility and an efficient response when adapting to the surroundings. However, there were notable differences in the modularity between the plants from F and X during their adaption to new environments. The plants from F displayed a higher level of trait aggregation, suggesting a greater degree of modularity in their functionality. This may be attributed to their prolonged exposure to homogeneous environments, where minimal disturbances allow for the development of stable patterns in which specific traits perform the same function over an extended period [7,35]. Consequently, this leads to the evolution of conservative functional operational patterns to maintain plant growth.

### 4.2. Characteristics of Plant Functional Traits in Different Treatments

The redundancy analysis divided all the samples into two groups based on the trophic level of the culture medium: one group consisted of samples cultivated in high-nutrient water, while the other group consisted of samples cultivated in low-nutrient water. The results indicate that the plant functional traits of *M. spicatum* are primarily influenced by the environment, rather than the plant source. The redundancy analysis results showed that the morphological indicators of plants, such as the leaf length, leaf number, plant height, and branch number have a significant positive correlation with the total phosphorus (TP), total nitrogen (TN), salinity (SAL), conductivity (C), and temperature (T) of the water. This suggests that environmental factors mainly influence the morphological characteristics of *M. spicatum*. In high-nutrient water bodies, *M. spicatum* exhibits a higher plant height and more branch numbers, and it also displays a greater leaf length and leaf number.

The main characteristics of eutrophic water bodies are excess nutrients and low transparency. Plants colonizing such environments struggle to obtain sufficient light energy for photosynthesis. Therefore, in order to meet their growth requirements, plants need to grow vigorously to approach the water surface and acquire enough light for photosynthesis. Increased branching, higher leaf numbers, and longer leaf length enables plants to occupy a wide range of ecological niches and gain a distinctive advantage in capturing light energy resources [45,46]. Under low light conditions, plants tend to lengthen their stems and leaves to acquire more light energy, while under high light intensity, they tend to reduce stem length and leaf area to avoid damage from excessive light [12,47]. Certainly, in addition to the factors mentioned above, other factors such as metals can also have a significant impact on plant traits. For example, Gałczyńska et al. [48] found that when nitrogen and phosphorus concentrations in water are low, the metal concentrations in the water can significantly affect the growth of *Hydrocharis morsus-ranae*.

### 4.3. Adaptation Strategies of Myriophyllum spicatum

Plant functional traits play a vital role in determining the adaptability of plants. The source of plants and the corresponding environmental conditions are crucial factors that influence these traits. Different sources give rise to variations in genetic diversity, stress resistance, and adaptability among plants. This, in turn, leads to differences in plant functional traits as they acclimate to their environment. Moreover, environmental factors in a specific habitat directly affect plant functional traits. Changes in light, temperature, and nutrient availability contribute to the distinct functional attributes observed in plant morphology, physiology, and ecology. Ultimately, these traits have a significant impact on plant growth, reproduction, and overall adaptability.

*M. spicatum* is a perennial submerged macrophyte with strong adaptability to various environments. One adaptation is its finely dissected leaves, which increase the surface area for efficient nutrient absorption from water [49]. Furthermore, *M. spicatum* exhibits a high tolerance to fluctuating water depths and nutrient availability, making it well-suited to diverse aquatic environments [50], including anaerobic sediments [51,52]. In this study, *M. spicatum* from two distinct sources exhibited successful adaptation to the new habitats. The results indicate that both plant source and environmental conditions have certain influences on the plants during their adaptation to a new environment. Specifically, the plant source solely affects the coordination patterns among plant functional traits, while the response of the plant functional traits to environmental factors holds more significance. Plants from Xingyun Lake tend to increase the connectivity among traits, thereby facilitating effective communication between traits and environmental factors. Their increased resource utilization and activation capacity enable them to adapt quickly to their environment. On the other hand, plants from Fuxian Lake tend to allow specific traits to perform specific functions, maintaining a stable and orderly operational pattern among different traits to successfully adapt to the new environment. Ultimately, under the influence of the environment, significant changes happen in the plant phenotypic traits to ensure the moderate acquisition of resources to maintain the normal operation of physiological functions. Therefore, the adaptation strategy of *M. spicatum* to the environment mainly relies on its phenotypic plasticity, which ensures a balanced acquisition of resources and promotes the stable and efficient operation of plant physiological traits.

## 5. Conclusions

The effects of both the plant source and environmental conditions in the habitat on the characteristics of plant functional traits were compared in this study using *M. spicatum* as the research material. The findings revealed that both the plant source and environment influenced plant functional traits. However, while the plant source primarily affected the coordination patterns among the plant functional traits, the environmental conditions in the habitat had a more pronounced impact on the plant functional traits. Specifically, this effect was predominantly observed in the morphological changes, while having minimal influence on the physiological traits. This may be due to environmental pressures not exceeding the tolerance threshold of *M. spicatum*, leading to a swift adaptation to the environment and achieving a relatively stable physiological state after one year of cultivation. *M. spicatum* is a highly suitable and extensively used plant in ecological freshwater restoration worldwide. It has a strong ability to resist high nutrient concentrations in freshwater ecosystems. In this study, *M. spicatum* mainly relied on modifying its phenotypic traits to maintain the stability of its physiological functions, thereby meeting the specific requirements under unique conditions. However, it is still unknown whether other species with high adaptability to harsh environments employ similar strategies in acclimatization. The results not only offered compelling evidence on the adaptation strategies of *M. spicatum* in variable environments, but also provided theoretical support for the conservation of biodiversity and sustainable development.

## Figures and Tables

**Table 1:** Physicochemical parameters of water in the four treatments. (n = 5).

Treatment	Date	T (°C)		C (μS/cm)		TDS (mg/L)		SAL (ppt)		pH		ORP (mV)		TN (mg/L)		TP (mg/L)	
		ave	sd	ave	sd	ave	sd	ave	sd	ave	sd	ave	sd	ave	sd	ave	sd
LF	August 2014	25.30	1.407	340.10	16.214	220.09	11.624	0.15	0.034	9.01	0.183	89.08	3.426	0.30	0.039	0.01	0.003
LX	August 2014	26.54	0.378	323.63	6.963	204.40	4.463	0.11	0.017	9.22	0.072	88.38	1.308	0.22	0.073	0.01	0.004
HF	August 2014	24.62	0.377	747.80	10.616	486.20	5.438	0.37	0.005	9.16	0.130	67.34	4.423	3.71	1.609	0.42	0.125
HX	August 2014	24.30	0.245	747.80	7.727	491.40	3.560	0.37	0.004	9.18	0.044	56.98	6.184	6.14	1.458	0.67	0.136
LF	August 2015	21.40	0.173	290.76	12.153	202.93	8.492	0.15	0.008	9.43	0.123	80.76	2.736	0.49	0.168	0.05	0.005
LX	August 2015	21.50	0.224	286.36	15.483	199.55	10.164	0.15	0.009	9.44	0.063	74.34	4.649	0.35	0.104	0.06	0.006
HF	August 2015	22.60	0.587	726.60	14.100	495.30	11.628	0.37	0.008	9.54	0.166	71.44	7.491	1.79	0.303	0.22	0.036
HX	August 2015	22.62	0.769	697.80	32.874	477.10	16.315	0.35	0.011	9.99	0.193	77.20	3.738	1.23	0.707	0.17	0.076

## Data Availability

Data are contained within the article.
